# Rapid review and meta-analysis of serial intervals for SARS-CoV-2 Delta and Omicron variants

**DOI:** 10.1186/s12879-023-08407-5

**Published:** 2023-06-26

**Authors:** Zachary J. Madewell, Yang Yang, Ira M. Longini, M. Elizabeth Halloran, Alessandro Vespignani, Natalie E. Dean

**Affiliations:** 1 Department of Biostatistics, University of Florida, Gainesville, FL USA; 2 Department of Statistics, University of Georgia, Athens, GA USA; 3 Department of Biostatistics, University of Washington, Seattle, WA USA; 4 Fred Hutchinson Cancer Center, Seattle, WA USA; 5 Laboratory for the Modeling of Biological and Socio-Technical Systems, Northeastern University, Boston, MA USA; 6 Department of Biostatistics and Bioinformatics, Emory University, Atlanta, GA USA

**Keywords:** Serial interval, Generation time, COVID-19, Symptom onset, Generation interval, Incubation period

## Abstract

**Background:**

The serial interval is the period of time between symptom onset in the primary case and symptom onset in the secondary case. Understanding the serial interval is important for determining transmission dynamics of infectious diseases like COVID-19, including the reproduction number and secondary attack rates, which could influence control measures. Early meta-analyses of COVID-19 reported serial intervals of 5.2 days (95% CI: 4.9–5.5) for the original wild-type variant and 5.2 days (95% CI: 4.87–5.47) for Alpha variant. The serial interval has been shown to decrease over the course of an epidemic for other respiratory diseases, which may be due to accumulating viral mutations and implementation of more effective nonpharmaceutical interventions. We therefore aggregated the literature to estimate serial intervals for Delta and Omicron variants.

**Methods:**

This study followed Preferred Reporting Items for Systematic Reviews and Meta-analyses guidelines. A systematic literature search was conducted of PubMed, Scopus, Cochrane Library, ScienceDirect, and preprint server medRxiv for articles published from April 4, 2021, through May 23, 2023. Search terms were: (“serial interval” or “generation time”), (“Omicron” or “Delta”), and (“SARS-CoV-2” or “COVID-19”). Meta-analyses were done for Delta and Omicron variants using a restricted maximum-likelihood estimator model with a random effect for each study. Pooled average estimates and 95% confidence intervals (95% CI) are reported.

**Results:**

There were 46,648 primary/secondary case pairs included for the meta-analysis of Delta and 18,324 for Omicron. Mean serial interval for included studies ranged from 2.3–5.8 days for Delta and 2.1–4.8 days for Omicron. The pooled mean serial interval for Delta was 3.9 days (95% CI: 3.4–4.3) (20 studies) and Omicron was 3.2 days (95% CI: 2.9–3.5) (20 studies). Mean estimated serial interval for BA.1 was 3.3 days (95% CI: 2.8–3.7) (11 studies), BA.2 was 2.9 days (95% CI: 2.7–3.1) (six studies), and BA.5 was 2.3 days (95% CI: 1.6–3.1) (three studies).

**Conclusions:**

Serial interval estimates for Delta and Omicron were shorter than ancestral SARS-CoV-2 variants. More recent Omicron subvariants had even shorter serial intervals suggesting serial intervals may be shortening over time. This suggests more rapid transmission from one generation of cases to the next, consistent with the observed faster growth dynamic of these variants compared to their ancestors. Additional changes to the serial interval may occur as SARS-CoV-2 continues to circulate and evolve. Changes to population immunity (due to infection and/or vaccination) may further modify it.

**Supplementary Information:**

The online version contains supplementary material available at 10.1186/s12879-023-08407-5.

## Introduction

The Delta SARS-CoV-2 variant (B.1.617) was first detected in India in October 2020 and Omicron variant (B.1.1.529) in South Africa in November 2021 [1]. They were designated the fourth and fifth variants of concern by the World Health Organization (WHO) due to their high transmissibility and ability to evade immune responses [1, 2]. Compared to the original wild-type variant, Omicron contains over 50 mutations, including 32 in the spike protein, that alters protein binding efficiency and immunogenicity, increasing infectivity, antibody escape ability, and the chance of reinfection [3]. Additional mutations led to multiple Omicron subvariants with increased transmissibility including BA.2, BA.2.12.1, BA.4, BA.5, BF.7, BQ.1, and XBB.1.5; as of May 2023 the latter accounted for most infections in the United States [4]. Compared with the Delta variant, there is evidence that Omicron replicates less efficiently in the lungs and more efficiently in the upper respiratory tract, which may contribute to increased transmissibility [5, 6]. Omicron also has a shorter incubation period (3.42 days; 95% CI: 2.88–3.96 days) compared to previous variants [7]. Lower hospitalization rates, shorter hospital stay, and lower case-fatality rates have been documented for Omicron compared to Delta, even after controlling for vaccination status [8].

The generation time (generation interval) is the time between infection of primary and secondary cases. Generation times are difficult to observe in practice and are often replaced with serial intervals, or the period of time between symptom onset in the primary case and symptom onset in the secondary case [9]. Generation time is never negative as the secondary case’s infection time always occurs after the primary case’s infection time, but serial interval for a primary/secondary case pair can be negative if the secondary case has symptom onset earlier than the primary case. Understanding the serial interval is important for determining transmission characteristics of infectious diseases like COVID-19, including the reproduction number and secondary attack rates, which in turn could influence the design of control measures [10–14]. The serial interval depends on both biological and sociological factors. Biological factors include the degree and duration of infectiousness of an index case, incubation period (time from infection to symptom onset), and latent period (time from infection to infectiousness) [15, 16]. Sociological factors include population contact patterns between infectious and susceptible individuals, which may vary concomitantly with public health interventions, lockdowns, and travel restrictions. The serial interval has been shown to decrease over the course of an epidemic [17], which may be due to accumulating viral mutations and/or implementation of more effective nonpharmaceutical interventions [18]. It is often difficult to estimate the serial interval when the pathogen is widespread in a population because of the uncertainties in linking primary and secondary cases. The predominant literature for COVID-19 serial interval focuses on the original wild-type and Alpha variants, with subsequent meta-analyses reporting serial intervals of 5.2 days for both variants [19–21]. A rapidly growing body of literature reports shorter serial intervals for Delta and Omicron variants. Here we expanded and aggregated those studies to estimate serial intervals for Delta and Omicron variants, which should help inform accurate estimation of important epidemiological quantities such as the reproductive number and more robust predictions using mathematical/statistical models.

## Methods

### Search strategy

This study followed Preferred Reporting Items for Systematic Reviews and Meta-analyses (PRISMA) guidelines. A systematic literature search was conducted of PubMed, Scopus, Cochrane Library, ScienceDirect, and preprint server medRxiv for articles published from April 4, 2021, when Delta was classified as a WHO Variant of Interest, through May 23, 2023. Search terms were: (“serial interval” or “generation time”), (“Omicron” or “Delta”), and (“SARS-CoV-2” or “COVID-19”) (Supplemental methods). Reference lists of selected papers were also screened for additional studies. There were no restrictions on language, study design, or place of publication. Preprints were included. Citations were managed in EndNote version 20 (Thomson Reuters).

### Eligibility criteria

All articles with original data for estimating clinical serial interval (time between symptom onset of primary and secondary case), diagnostic serial interval (time between diagnosis dates of primary and secondary case), or generation time (time between infection of primary and secondary case) were included. Studies were included if they reported mean serial interval or generation time and standard deviation for primary/secondary case pairs. Excluded studies 1) were done before the emergence of Delta or Omicron and 2) only reported mean or median serial interval without standard deviations (SD). Studies that did not report mean serial intervals or SDs were included, however, if they provided the underlying serial interval data, which we used to estimate the serial interval distribution and SDs (see Statistical Analysis section). One reviewer (Z.J.M.) first screened studies by titles and abstracts to identify potential studies for inclusion. That reviewer then evaluated full-text articles and selected those that met the inclusion criteria.

### Data extraction

For this study, one reviewer (Z.J.M.) extracted the following information: first author, location, article type, primary case symptom onset dates, SARS-CoV-2 variant, contact setting, statistical distribution, serial interval or generation time, study adjusted for right truncation or not (i.e., secondary cases that were not yet detected at the time of the study were excluded from the data set), number of primary/secondary case pairs, mean serial interval, and SD.

### Evaluation of study quality and risk of bias

We used the Newcastle–Ottawa Scale (NOS) to assess the methodological quality and risk of bias of included studies [22]. We used an adapted version of the NOS for cross-sectional studies designed by Herzog et al. [23]. Studies could earn up to 10 points in participant selection (maximum 5 stars), study comparability (maximum 2 stars), and outcome of interest (maximum 3 stars). Studies were classified as having high (≤ 3 stars), moderate (4–6 stars), and low (≥ 7 stars) risk of bias. One of us (Z.J.M.) evaluated the study quality and assigned the quality grades. We also used funnel plots, Egger’s test, and Begg and Mazumdar rank correlation to evaluate the potential for publication bias, with significance set at *P* < 0.10 [24, 25].

### Statistical analysis

For studies that did not report mean serial interval or SD, but provided the raw serial interval data, we used EpiEstim package [26] in R software version 4.2.3 (R Project for Statistical Computing) to fit log-normal, Weibull, and gamma distributions to the raw difference-in-days data, and subsequently calculated means and SDs [26]. Akaike’s information criterion (AIC) was used to compare fits, and the model with the lowest AIC value was selected. If a study reported a skewed normal distribution but the mean or SD was not provided, we used maximum likelihood estimation to fit a skewed normal distribution to the raw serial interval data using the package sn in R [27].

Overall meta-analyses were done for Delta and Omicron using a restricted maximum-likelihood estimator model with a random effect for each study. The Cochran Q test and *I*^*2*^ statistic are reported as measures of statistical heterogeneity. *I*^*2*^ values of 25%, 50%, and 75% indicated low, moderate, and high heterogeneity, respectively. Meta-analyses were done using metafor package in R [28]. Pooled average estimates and 95% confidence intervals (95% CI) are shown in forest plots. We conducted sensitivity analyses 1) excluding generation time studies, 2) restricted to studies in which serial intervals were reported for both Delta and Omicron variants to control for between-study heterogeneity, and 3) restricted to studies at low risk of bias from the NOS assessment. We further evaluated serial intervals by Omicron subvariant and for Delta and Omicron disaggregated by transmission setting (household/community).

## Results

**Fig. 1:**
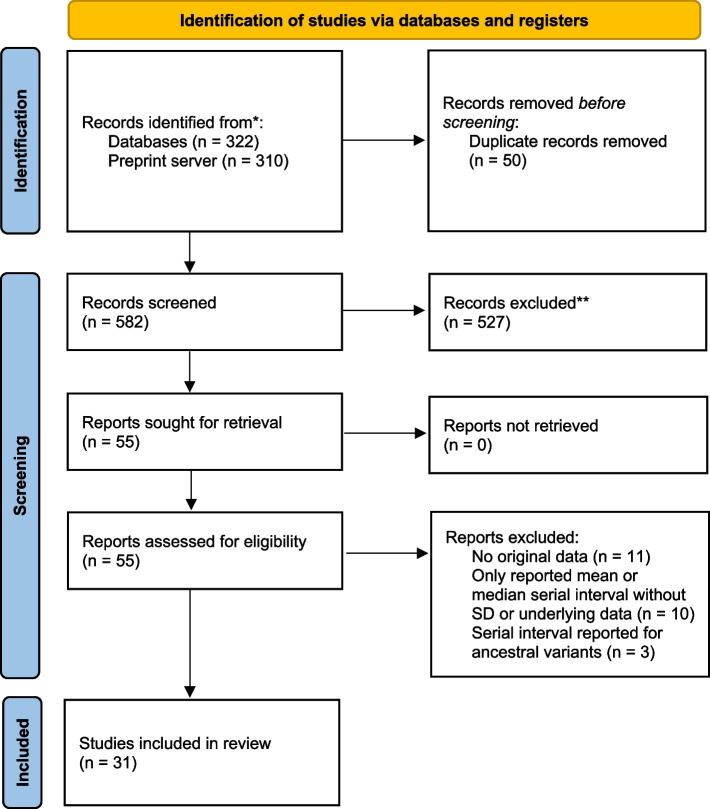
PRISMA Flow Diagram

Our search retrieved 582 deduplicated records between April 4, 2021, and May 23, 2023 (Fig. 1). Thirty-one studies [29–59] were included in this review (Table S1). Studies included 17 research articles [29, 32, 34, 35, 40, 42, 43, 45–48, 50, 53–55, 57, 59], eight brief communications [30, 31, 33, 36, 39, 51, 52, 58], three letters [37, 38, 41], and three reports [44, 49, 56]. Studies were from Belgium [33], Brazil [37], China [34–36, 42, 44, 45, 49–51, 53–57, 59], Germany [29], Japan [46], Netherlands [30], Singapore [38, 52], South Korea [32, 39–41, 58], Spain [31], U.K. [47], and U.S.A. [43, 48]. Twenty studies were at low risk of bias and 11 moderate, primarily due to small sample sizes (Table S2).

Distributions used to fit the serial interval data were gamma (15 studies [29, 36, 44–47, 52–59]), normal (five studies [32–34, 39, 40]), Weibull (three studies [42, 49, 50]), Gaussian (one study [37]), and skewed normal (one study [38]); the distribution was unspecified for four studies [30, 31, 35, 41]. All studies but four reported mean and SD for serial interval. Two studies reported the mean serial interval by fitting skewed normal [38] and gamma distributions [52], but did not provide SD. Using the raw difference-in-days data, we fit skewed normal and gamma distributions to obtain SDs. Two other studies did not specify the distribution and did not report mean serial interval or SD but provided the raw serial interval data which included only positive values [48]. The log-normal distribution provided the best fit to the serial interval data for both studies. Only two studies [47, 57] reported mean generation time and two studies [51, 54] reported adjusting for right truncation. Nine studies [29, 30, 38, 41, 42, 47, 48, 50, 58] were exclusively from the household setting. The study with the largest sample size [29] only reported the number of households included in the serial interval analysis rather than the number of primary/secondary case pairs. The study included 39,277 households for Delta and 11,512 for Omicron and reported that 31% of households comprised only two cases. We assumed each household represented a case pair, therefore the n used in this meta-analysis was an underestimate for that study.

**Fig. 2:**
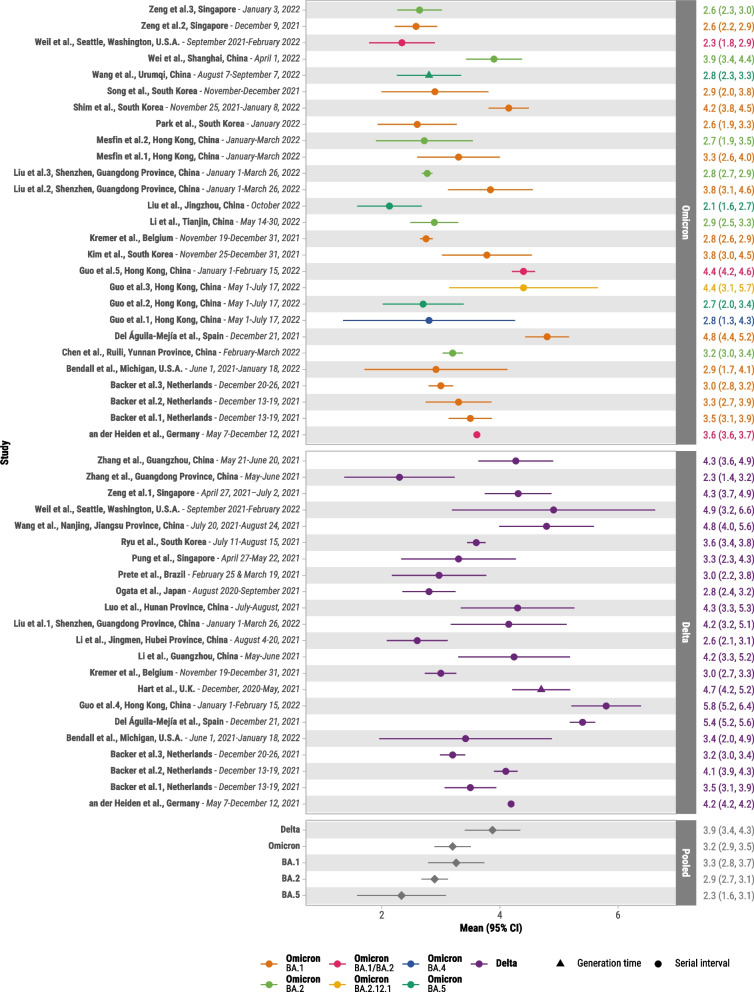
Forest plot of serial interval estimates for Delta and Omicron variants. Primary case symptom onset dates are provided for each study. Mean serial intervals and 95% confidence intervals are shown on the right

There were 46,648 case pairs included for the meta-analysis of Delta and 18,324 for Omicron. Mean serial interval for included studies ranged from 2.3 days [44] to 5.8 days [31, 54] for Delta and 2.1 days [43, 59] to 4.8 days [31] for Omicron. The pooled mean serial interval for Delta was 3.9 days (95% CI: 3.4–4.3) (20 studies [29–31, 33–35, 37–39, 42–49, 52–54]) and Omicron was 3.2 days (95% CI: 2.9–3.5) (20 studies [29–33, 36, 40, 41, 43, 48, 50–59]) (Fig. 2). Moderate heterogeneity was found for Delta (*I*^*2*^ = 73.5%; *P* < 0.001) and Omicron (*I*^*2*^ = 64.1%; *P* < 0.001) estimates. Publication bias was not suspected for studies of Delta or Omicron (Figure S1). Excluding studies [47, 57] that reported generation time, mean serial interval for Delta was 3.8 days (95% CI: 3.4–4.2) and Omicron was 3.2 days (95% CI: 2.9–3.5). Restricting to nine studies [29–31, 33, 43, 48, 52–54] that reported serial intervals for both Delta and Omicron, the pooled mean serial interval for Delta was 4.2 days (95% CI: 3.5–4.9) and Omicron was 3.2 days (95% CI: 2.7–3.8) (Figure S2). Restricting to studies at low risk of bias, mean serial interval for Delta was 4.0 days (95% CI: 3.5–4.5) (14 studies [29–31, 33, 34, 38, 39, 42, 46–49, 52, 54]) and Omicron was 3.3 days (95% CI: 2.9–3.7) (14 studies [29–31, 33, 36, 40, 48, 50–54, 56, 57]).

Examining specific Omicron subvariants, mean serial interval was 3.3 days (95% CI: 2.8–3.7) for BA.1 (11 studies [30–33, 36, 40, 41, 48, 52, 53, 58]), 2.9 days (95% CI: 2.7–3.1) for BA.2 (6 studies [36, 50, 52, 53, 55, 56]), and 2.3 days (95% CI: 1.6–3.1) for BA.5 (3 studies [51, 57, 59]). Excluding one study of generation time [57], serial interval for BA.5 was 2.3 days (95% CI: 1.4–3.1). One study [51] reported mean estimates for BA.4 of 2.8 days (95% CI: 1.5–6.7) and BA.2.12.1 of 4.4 days (95% CI: 2.6–7.5). Estimated mean serial interval was 3.2 and 3.1 days for household and community transmission for Omicron, whereas mean serial interval was slightly shorter for the community setting for Delta (3.6 days, 95% CI: 3.0–4.2) than the household setting (4.2 days, 95% CI: 4.1–4.3) (Figure S3).

## Discussion

Estimated serial intervals for Delta (3.9 days, 95% CI: 3.4–4.3) and Omicron (3.2 days, 95% CI: 2.9–3.5) were shorter than serial intervals reported for Alpha (5.2 days, 95% CI: 4.87–5.47) [21] and the original wild-type variant (5.2 days, 95% CI: 4.9–5.5 [19]; and 5.2 days, 95% CI: 4.4–6.0 [20]). Our estimates were also shorter than serial intervals for respiratory syncytial virus (7.5 days), severe acute respiratory syndrome (SARS) (8.4 days), and Middle East Respiratory Syndrome (MERS) (12.6 days), but longer than the serial interval for influenza A(H3N2) (2.2 days) [60, 61]. Mean serial interval for Omicron subvariants BA.2 (2.9 days, 95% CI: 2.7–3.1) and BA.5 (2.3 days, 95% CI: 1.6–3.1) were shorter than that of BA.1 (3.3 days, 95% CI: 2.8–3.7), suggesting serial intervals may be shortening over time. A recent conference abstract that was not included in this analysis (it did not report SD, number of case pairs, and did not provide the underlying data) of the Virus Watch study in England and Wales reported an even shorter mean serial interval for the Omicron BA.5 subvariant of 2.02 days (95% CrI: 1.26–2.84) [62]. In the absence of vaccines, the shorter the serial interval of the virus the more difficult it is to mitigate the rapid generation of secondary cases [63].

Shorter serial intervals also affect the estimation of epidemic transmissibility parameters such as the effective reproduction number, *R*_*t*_, defined as the average number of individuals infected by a single infected case in a large population on day *t*. Real-time estimation of *R*_*t*_ is important for evaluating the effectiveness of public health measures (e.g., vaccination, isolation, quarantine) and determining whether current public health measures need to be intensified [64–66]. One study demonstrated that modeling with time-varying serial intervals more accurately estimated *R*_*t*_ compared to a single static serial interval, and advised using caution when applying serial interval estimates to different settings and time periods [18]. A meta-analysis reported pooled effective reproduction numbers for Omicron subvariants BA.1 and BA.2 of 3.22 (95% CI: 2.31–4.14) and 5.04 (95% CI: 4.33–5.75); the review included one study for BA.5 which reported a *R*_*t*_ of 5.22 (95% credible interval: 4.65–5.79) [67]. Serial intervals can become shorter during the course of an epidemic due to the synergy of multiple factors, e.g., behavioral changes, case isolation, improved contact tracing systems, nonpharmaceutical interventions, viral mutations, and susceptible depletion among close contacts [17, 63, 68–70]. The increasing growth rate and more rapid transmission cycles mathematically associated with shortened serial intervals can challenge the healthcare system since contact tracing needs to keep up with the rapid replacement of case generations.

Estimated serial interval for Delta was slightly shorter than the incubation period reported for Delta (4.4 days; 95% CI, 3.8–5.1 days) from a systematic review and meta-analysis, whereas serial interval for Omicron was nearly identical to the incubation period (3.4 days; 95% CI, 2.9–4.0 days) [7]. When the serial interval is shorter than the incubation period, that suggests pre-symptomatic transmission has occurred, whereas serial intervals longer than the incubation period suggest most transmission occurred after symptom onset in a primary case [71]. Our systematic review included different studies than the review of incubation period, so the estimates are not directly comparable because of between-study heterogeneity. Serial intervals strongly depend on human behavior and can decrease concomitant with increasing interventions [18]. We found a slightly shorter serial interval for community transmission of Delta than household transmission, which could be attributed to improved nonpharmaceutical interventions such as rapid isolation of cases and thorough contact tracing [18, 72].

This study had several limitations. First, there was moderate heterogeneity for Delta and Omicron serial interval estimates, which may be attributed to differences in study design, study period, infection incidence, population characteristics, human behavior, and analytic methods. Several studies fitted positive distributions like Gamma and Weibull that do not include negative serial intervals. However, seven studies [32, 36, 46, 49–51, 54] with non-positive serial interval data reported shifting the data by adding several days to each serial interval in order to fit the Gamma or Weibull distributions. Second, serial interval may vary by age, comorbidity status, vaccination status, or other covariates, but that data was not reported in most studies precluding meta-analysis. For example, one study included in this review found the mean serial interval for Omicron to be two days shorter from child primary cases than adult primary cases which may be associated with lower vaccination uptake in children or behavioral factors [32]. Third, precise ascertainment of symptom onset dates is critical for serial interval estimation, but initial COVID-19 symptoms can be non-specific and unrelated to SARS-CoV-2 infection. Fourth, consistent with other systematic reviews of serial intervals for SARS-CoV-2 [19, 20], our analysis only included clinical serial intervals characterized by symptom onset dates. Our estimates for serial intervals thus may not reflect generation times involving asymptomatic primary cases, as studies suggest viral shedding from asymptomatic carriers may differ from symptomatic carriers [73]. Disregarding the time scale of asymptomatic transmission can bias reproduction number estimation [74]. More specifically, *R*_*0*_ will be overestimated if asymptomatic cases have shorter generation intervals than symptomatic cases, whereas *R*_*0*_ will be underestimated if they have longer generation intervals [74]. A possible alternative is to use diagnostic serial intervals, or the time between diagnosis dates of primary and secondary cases, which are defined for asymptomatic cases and have been proposed as a more objective measure than onset of symptoms [69]. Nevertheless, none of the studies we collected contain data on diagnostic serial intervals. Fifth, despite studies reporting careful selection of linked primary/secondary case pairs, exposure from additional unknown or asymptomatic sources may have occurred. Sixth, only two included studies reported adjusting for right truncation—the selection bias such that cases with shorter incubation periods are more likely to be included in the study. Seventh, our study included BA.1, BA.2, BA.2.12.1, BA.4, and BA.5 Omicron subvariants, but more recent subvariants may have different serial intervals. Eighth, to increase speed of the review process, a single reviewer was responsible for title/abstract screening, full text screening, and the risk of bias assessment, consistent with other rapid reviews [75]. Having a second reviewer independently screen and review articles may have identified additional studies for inclusion. Notwithstanding these limitations, we are unaware of other systematic reviews focusing on serial intervals for Delta or Omicron variants.

Serial interval estimates for Omicron were shorter than ancestral SARS-CoV-2 variants, which may reduce the effectiveness of public health interventions like contact tracing. Additional changes to the serial interval may occur as SARS-CoV-2 continues to circulate and evolve. Changes to population immunity (due to infection and/or vaccination) may further modify it.

## Supplementary Information

**Additional file 1.**

## Data Availability

All relevant data are within the manuscript.

## Abbreviations

AIC: Akaike’s information criterion
PRISMA: Preferred Reporting Items for Systematic Reviews and Meta-analyses
SD: Standard deviation
WHO: World Health Organization
