# Supplementary material for: Rapid review and meta-analysis of serial intervals for SARS-CoV-2 Delta and Omicron variants
Source: BMC Infect Dis. 2023 Jun 26;23:429. doi: 10.1186/s12879-023-08407-5 (PMC10291789; doi:10.1186/s12879-023-08407-5)
Supplement: Supplementary file 1 — Additional file 1. [file 12879_2023_8407_MOESM1_ESM.docx]

**Additional File 1.**

**Supplemental Methods**

*Search Strategy*

Publication time: April 4, 2021, to May 23, 2023

Key words:

#1 (SARS-CoV-2) OR (COVID-19)

#2 (Omicron) OR (Delta)

#3 (serial interval) OR (generation time)

| Database | Number of results |
| --- | --- |
| PubMed | 45 |
| Scopus | 52 |
| Cochrane Library | 0 |
| ScienceDirect | 225 |
| #1 AND #2 AND #3 | |

Preprint database used different searching rules and limitations

| Database | Number of results |
| --- | --- |
| medRxiv | 310 |
| for term "SARS-CoV-2 COVID-19 Omicron Delta serial interval" and posted between "April 4, 2021, to May 23, 2023" | |

**Table S1**. Description of studies identified from April 4, 2021, to May 23, 2023.

| Authors | Location | Article Type | Dates | Variant | Subvariant | Setting | Distribution | Adjusted for right truncation | Serial interval or generation time | Pairs | Mean | SD |
| --- | --- | --- | --- | --- | --- | --- | --- | --- | --- | --- | --- | --- |
| an der Heiden et al. (29) | Germany | Letter | May 7-December 12, 2021 | Delta | Delta | Household | Gamma | No | SI | 39277^a^ | 4.19 | 0.02 |
| an der Heiden et al. (29) | Germany | Letter | May 7-December 12, 2021 | Omicron | BA.1/BA.2 | Household | Gamma | No | SI | 11512^a^ | 3.61 | 0.03 |
| Backer et al. (30) | Netherlands | Brief communication | December 13-19, 2021 | Omicron | BA.1 | Household | Unspecified | No | SI | 164 | 3.5 | 2.4 |
| Backer et al. (30) | Netherlands | Brief communication | December 13-19, 2021 | Omicron | BA.1 | Community | Unspecified | No | SI | 71 | 3.3 | 2.4 |
| Backer et al. (30) | Netherlands | Brief communication | December 20-26, 2021 | Omicron | BA.1 | Household | Unspecified | No | SI | 480 | 3 | 2.3 |
| Backer et al. (30) | Netherlands | Brief communication | December 13-19, 2021 | Delta | Delta | Community | Unspecified | No | SI | 158 | 3.5 | 2.8 |
| Backer et al. (30) | Netherlands | Brief communication | December 13-19, 2021 | Delta | Delta | Household | Unspecified | No | SI | 761 | 4.1 | 2.8 |
| Backer et al. (30) | Netherlands | Brief communication | December 20-26, 2021 | Delta | Delta | Community | Unspecified | No | SI | 572 | 3.2 | 2.6 |
| Bendall et al. (48) | Michigan, U.S.A. | Research article | June 1, 2021-January 18, 2022 | Delta | Delta | Household | Estimated Log-normal distribution from raw data | No | SI | 10 | 3.41 | 2.36 |
| Bendall et al. (48) | Michigan, U.S.A. | Research article | June 1, 2021-January 18, 2022 | Omicron | BA.1 | Household | Estimated Log-normal distribution from raw data | No | SI | 17 | 2.92 | 2.55 |
| Chen et al. (56) | Ruili City, Yunnan Province, China | Report | February-March 2022 | Omicron | BA.2 | Community | Gamma | No | Si | 387 | 3.2 | 1.7 |
| Del Águila-Mejía et al. (31) | Spain | Brief communication | December 21, 2021 | Omicron | BA.1 | Community | Unspecified | No | SI | 252 | 4.8 | 3.0 |
| Del Águila-Mejía et al. (31) | Spain | Brief communication | December 21, 2021 | Delta | Delta | Community | Unspecified | No | SI | -- | 5.4 | 3.1 |
| Guo et al. (51) | Hong Kong, China | Brief communication | May 1-July 17, 2022 | Omicron | BA.4 | Community | Gamma | Yes | SI | 8 | 2.8 | 2.1 |
| Guo et al. (51) | Hong Kong, China | Brief communication | May 1-July 17, 2022 | Omicron | BA.5 | Community | Gamma | Yes | SI | 51 | 2.7 | 2.5 |
| Guo et al. (51) | Hong Kong, China | Brief communication | May 1-July 17, 2022 | Omicron | BA.2.12.1 | Community | Gamma | Yes | SI | 45 | 4.4 | 4.3 |
| Guo et al. (54) | Hong Kong, China | Research article | January 1-February 15, 2022 | Delta | Delta | Community | Gamma | Yes | SI | 168 | 5.8 | 3.9 |
| Guo et al. (54) | Hong Kong, China | Research article | January 1-February 15, 2022 | Omicron | BA.1/BA.2 | Community | Gamma | Yes | SI | 1090 | 4.4 | 3.3 |
| Hart et al. (47) | U.K. | Research article | December, 2020-May, 2021 | Delta | Delta | Household | Gamma | No | GT | 174 | 4.7 | 3.3 |
| Kim et al. (32) | South Korea | Research article | November 25-December 31, 2021 | Omicron | BA.1 | Community | Normal | No | SI | 73 | 3.78 | 3.33 |
| Kremer et al. (33) | Belgium | Brief communication | November 19-December 31, 2021 | Omicron | BA.1 | Community | Normal | No | SI | 2161 | 2.75 | 2.53 |
| Kremer et al. (33) | Belgium | Brief communication | November 19-December 31, 2021 | Delta | Delta | Community | Normal | No | SI | 334 | 3 | 2.48 |
| Li et al. (34) | Guangzhou, China | Research article | May-June 2021 | Delta | Delta | Community | Normal | No | SI | 67 | 4.24 | 3.95 |
| Li et al. (35) | Jingmen, Hubei Province, China | Research article | August 4-20, 2021 | Delta | Delta | Community | Unspecified | No | SI | 58 | 2.6 | 2.0 |
| Li et al. (55) | Tianjin, China | Research article | May 14-30, 2022 | Omicron | BA.2 | Community | Gamma | No | Si | 21 | 2.89 | 0.95 |
| Liu et al. (53) | Shenzhen, Guangdong Province, China | Research article | January 1-March 26, 2022 | Delta | Delta | Community | Gamma | No | SI | 16 | 4.15 | 2.0 |
| Liu et al. (53) | Shenzhen, Guangdong Province, China | Research article | January 1-March 26, 2022 | Omicron | BA.1 | Community | Gamma | No | SI | 34 | 3.84 | 2.3 |
| Liu et al. (53) | Shenzhen, Guangdong Province, China | Research article | January 1-March 26, 2022 | Omicron | BA.2 | Community | Gamma | No | SI | 1064 | 2.77 | 1.6 |
| Liu et al. (59) | Jingzhou, China | Research article | October 2022 | Omicron | BA.5 | Community | Gamma | No | SI | 37 | 2.13 | 1.69 |
| Luo et al. (49) | Hunan Province, China | Report | July-August, 2021 | Delta | Delta | Community | Weibull | No | SI | 54 | 4.3 | 3.6 |
| Mesfin et al. (36) | Hong Kong, China | Brief communication | January-March 2022 | Omicron | BA.1 | Community | Gamma | No | SI | 30 | 3.30 | 1.95 |
| Mesfin et al. (36) | Hong Kong, China | Brief communication | January-March 2022 | Omicron | BA.2 | Community | Gamma | No | SI | 13 | 2.72 | 1.51 |
| Ogata et al. (46) | Japan | Research article | August 2020-September 2021 | Delta | Delta | Community | Gamma | No | SI | 88 | 2.8 | 2.15 |
| Park et al. (58) | South Korea | Brief communication | January, 2022 | Omicron | BA.1 | Household | Gamma | No | SI | 31 | 2.6 | 1.9 |
| Prete et al. (37) | Brazil | Letter | February 25 & March 19, 2021 | Delta | Delta | Community | Gaussian | No | SI | 65 | 2.97 | 3.29 |
| Pung et al. (38) | Singapore | Letter | April 27-May 22, 2021 | Delta | Delta | Household | Skewed normal | No | SI | 32 | 3.3 | 2.8 |
| Ryu et al. (39) | South Korea | Brief communication | July 11-August 15, 2021 | Delta | Delta | Community | Normal | No | SI | 3728 | 3.6 | 4.9 |
| Shim et al. (40) | South Korea | Research article | November 25, 2021-January 8, 2022 | Omicron | BA.1 | Community | Normal | No | SI | 202 | 4.15 | 2.48 |
| Song et al. (41) | South Korea | Letter | November-December 2021 | Omicron | BA.1 | Household | Unspecified | No | SI | 12 | 2.9 | 1.6 |
| Wang et al. (42) | Nanjing, Jiangsu Province, China | Research article | July 20, 2021-August 24, 2021 | Delta | Delta | Household | Weibull | No | SI | 72 | 4.79 | 3.47 |
| Wang et al. (57) | Urumqi, China | Research article | August 7-September 7, 2022 | Omicron | BA.5 | Community | Gamma | No | GT | 178 | 2.8 | 3.7 |
| Wei et al. (50) | Shanghai, China | Preprint | April 1, 2022 | Omicron | BA.2 | Household | Weibull | No | SI | 234 | 3.9 | 3.7 |
| Weil et al. (43) | Seattle, Washington, U.S.A. | Research article | September 2021-February 2022 | Delta | Delta | Community | Estimated Log-normal distribution from raw data | No | SI | 8 | 4.91 | 2.48 |
| Weil et al. (43) | Seattle, Washington, U.S.A. | Research article | September 2021-February 2022 | Delta | Delta | Community | Estimated Log-normal distribution from raw data | No | SI | 43 | 2.34 | 1.86 |
| Zeng et al. (52) | Singapore | Brief communication | April 27, 2021–July 2, 2021 | Delta | Delta | Community | Gamma | No | SI | 88 | 4.31 | 2.70 |
| Zeng et al. (52) | Singapore | Brief communication | December 9, 2021 | Omicron | BA.1 | Community | Gamma | No | SI | 76 | 2.58 | 1.60 |
| Zeng et al. (52) | Singapore | Brief communication | January 3, 2022 | Omicron | BA.2 | Community | Gamma | No | SI | 38 | 2.64 | 1.19 |
| Zhang et al. (44) | Guangdong Province, China | Report | May-June 2021 | Delta | Delta | Community | Gamma | No | SI | 51 | 2.3 | 3.4 |
| Zhang et al. (45) | Guangzhou, China | Research article | May 21-June 20, 2021 | Delta | Delta | Community | Gamma | No | SI | 67 | 4.27 | 2.65 |
| ^a^ This study only reported the number of households included in the serial interval analysis rather than the number of primary/secondary case pairs. The study included 39,277 households for Delta and 11,512 for Omicron and reported that 31% of households comprised only two cases. We assumed each household represented a case pair, therefore the n was an underestimate for that study. | | | | | | | | | | | | |

**Table S2**. Risk of bias assessment for studies included in review

| Study | Selection | | | | Comparability | Outcome | | | Total score (maximum 10 stars) | Risk of bias |
| --- | --- | --- | --- | --- | --- | --- | --- | --- | --- | --- |
|  | Representativeness of the sample | Sample Size | Non-response rate | Ascertainment of the exposure | Confounding factors are controlled or investigated by subanalysis | | Assessment of the outcome | Statistical test |  |  |
| an der Heiden et al. (29) | ++ | + | + | + | + | | ++ | + | 9 | Low |
| Backer et al. (30) | ++ | + | + | + | ++ | | ++ |  | 9 | Low |
| Bendall et al. (48) | ++ |  | + | + | + | | ++ |  | 7 | Low |
| Chen et al. (56) | ++ | + | + | + |  | | + | + | 7 | Low |
| Del Águila-Mejía et al. (31) | ++ | + | + | + | + | | ++ |  | 8 | Low |
| Hart et al. (47) | + | + | + | + | + | | ++ | + | 8 | Low |
| Guo et al. (51) | + |  | + | + | ++ | | ++ | + | 8 | Low |
| Guo et al. (54) | ++ | + | + | + | ++ | | + | + | 9 | Low |
| Kim et al. (32) | + |  | + | + |  | | ++ | + | 6 | Moderate |
| Kremer et al. (33) | ++ | + | + | + | + | | ++ | + | 9 | Low |
| Li et al. (34) | + |  | + | + | + | | ++ | + | 7 | Low |
| Li et al. (35) | + |  | + | + |  | | ++ |  | 5 | Moderate |
| Li et al. (55) | + |  | + | + |  | | ++ | + | 6 | Moderate |
| Liu et al. (53) | + | + | + | + | + | | ++ | + | 8 | Low |
| Liu et al. (59) | + |  | + | + |  | | + | + | 5 | Moderate |
| Luo et al. (49) | + |  | + | + |  | | ++ | + | 6 | Moderate |
| Mesfin et al. (36) | + |  | + | + | + | | ++ | + | 7 | Low |
| Ogata et al. (46) | + |  | + | + | + | | ++ | + | 7 | Low |
| Park et al. (58) | + |  | + | + |  | | ++ | + | 6 | Moderate |
| Prete et al. (37) | + |  | + | + |  | | + | + | 5 | Moderate |
| Pung et al. (38) | + |  | + | + | + | | ++ | + | 6 | Low |
| Ryu et al. (39) | ++ | + | + | + | + | | ++ | + | 9 | Low |
| Shim et al. (40) | + | + | + | + |  | | ++ | + | 7 | Low |
| Song et al. (41) |  |  | + | + |  | | ++ |  | 4 | Moderate |
| Wang et al. (42) | + |  | + | + | ++ | | ++ | + | 8 | Low |
| Wang et al. (57) | ++ | + | + | + | ++ | | ++ | + | 10 | Low |
| Wei et al. (50) | ++ | + | + | + |  | | ++ | + | 8 | Low |
| Weil et al. (43) | + |  | + | + | + | | ++ |  | 6 | Moderate |
| Zeng et al. (52) | + |  | + | + | ++ | | ++ | + | 8 | Low |
| Zhang et al. (44) | + |  | + | + |  | | + | + | 5 | Moderate |
| Zhang et al. (45) | + |  | + | + |  | | ++ | + | 6 | Moderate |

**Figure S1**. Funnel plots of studies reporting mean serial intervals for Delta and Omicron SARS-CoV-2 variants.


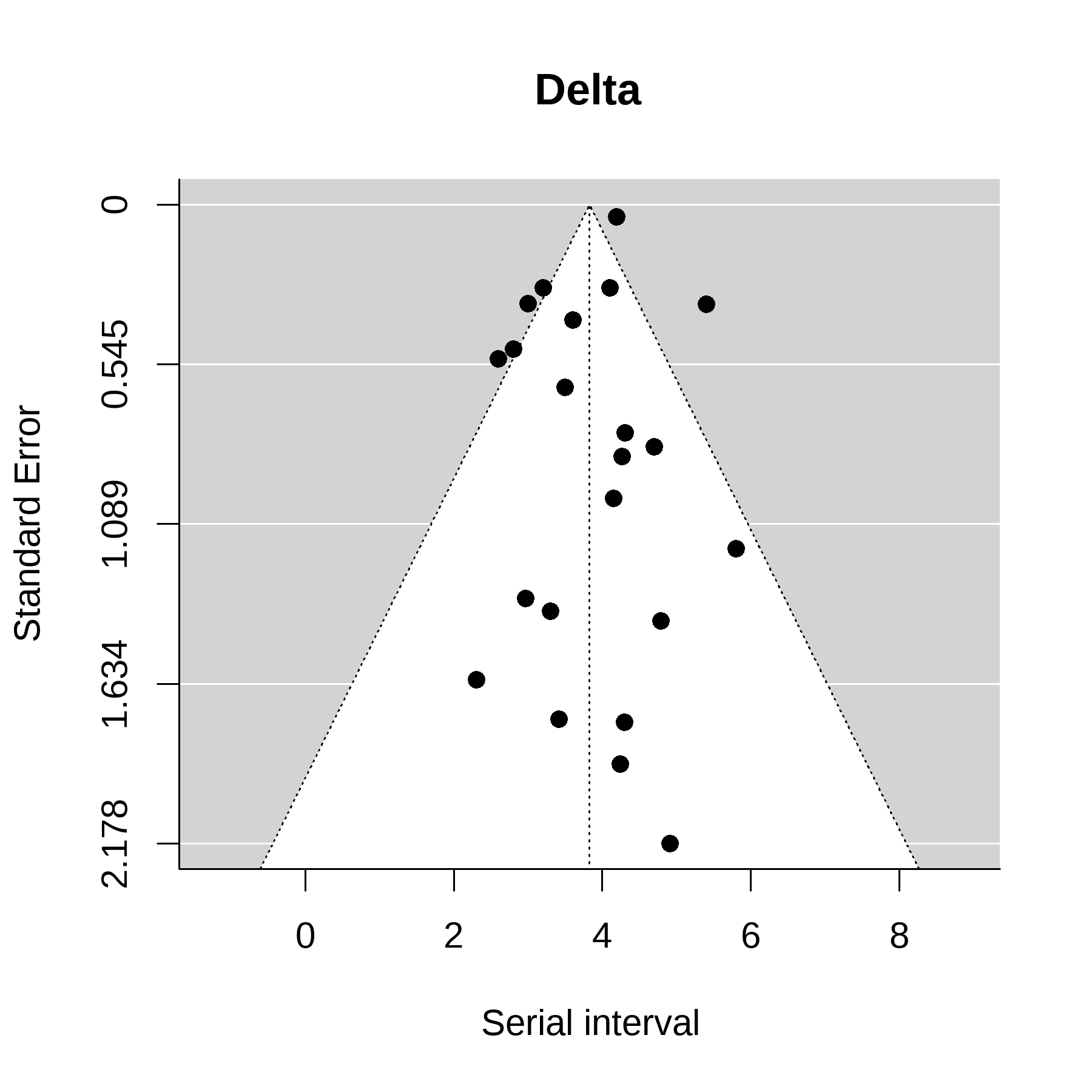

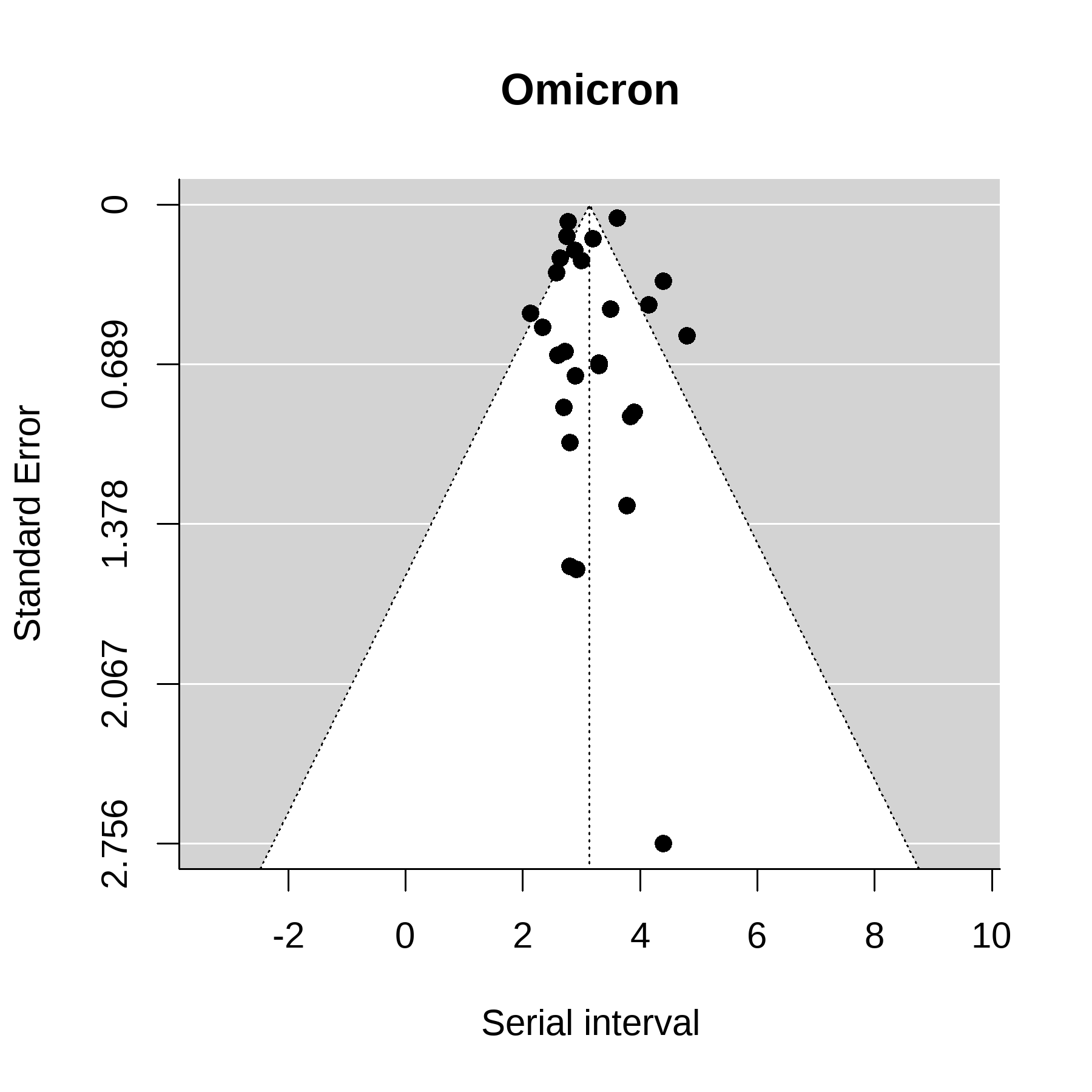


**Delta**

Egger’s test: z = 0.322, p = 0.747

Begg and Mazumdar rank correlation: Kendall's tau = 0.169, p = 0.288

**Omicron**

Egger’s test: z = 0.552, p = 0.581

Begg and Mazumdar rank correlation: Kendall's tau = 0.213, p = 0.142

**Figure S2**. Forest plot of serial interval estimates for Delta and Omicron variants restricted to studies that reported serial interval for both Delta and Omicron. Primary case symptom onset dates are provided for each study. Mean serial intervals and 95% confidence intervals are shown on the right.

**
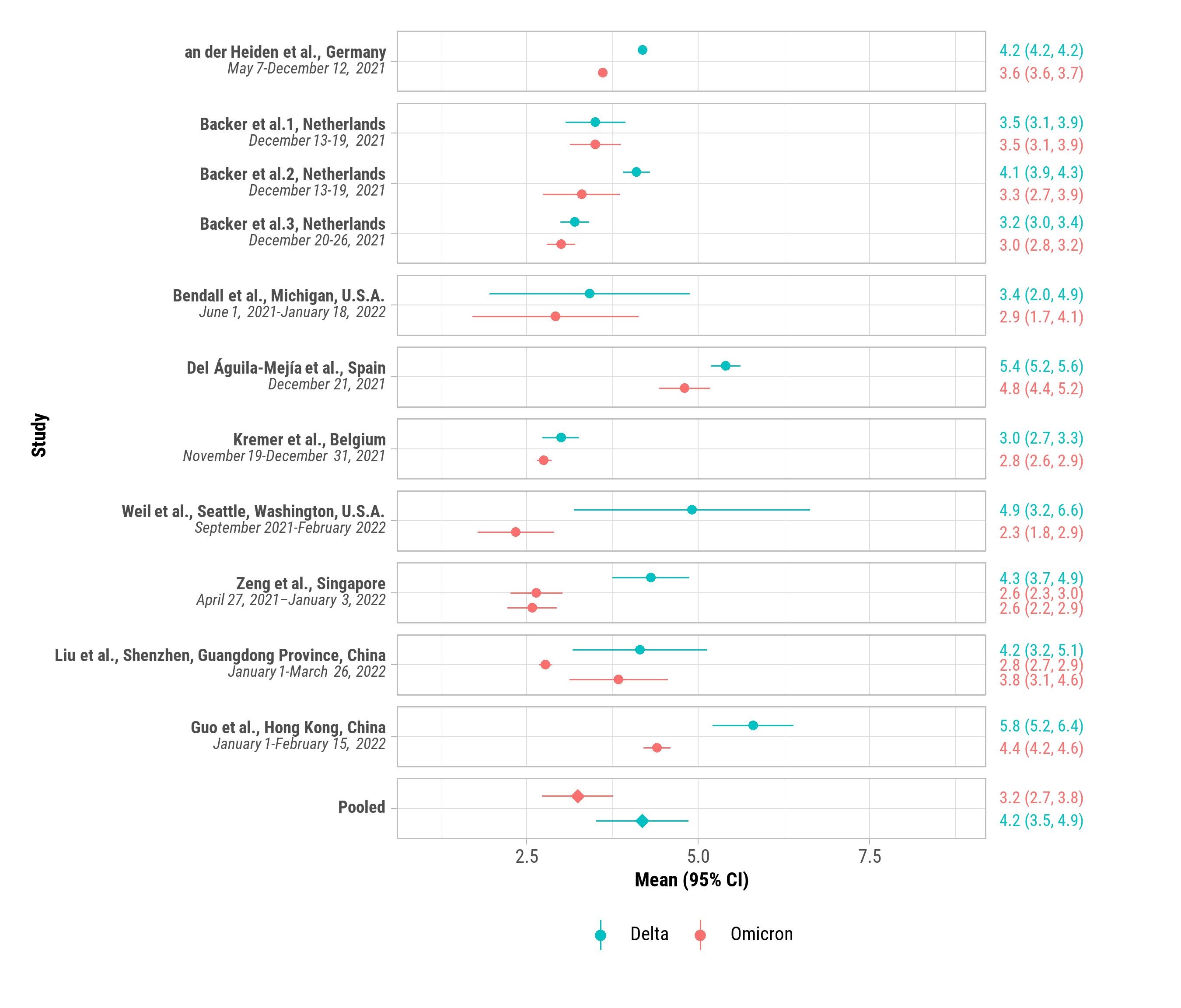
Figure S3**. Forest plot of serial interval estimates for Delta and Omicron variants by household/community transmission setting. Primary case symptom onset dates are provided for each study. Mean serial intervals and 95% confidence intervals are shown on the right.


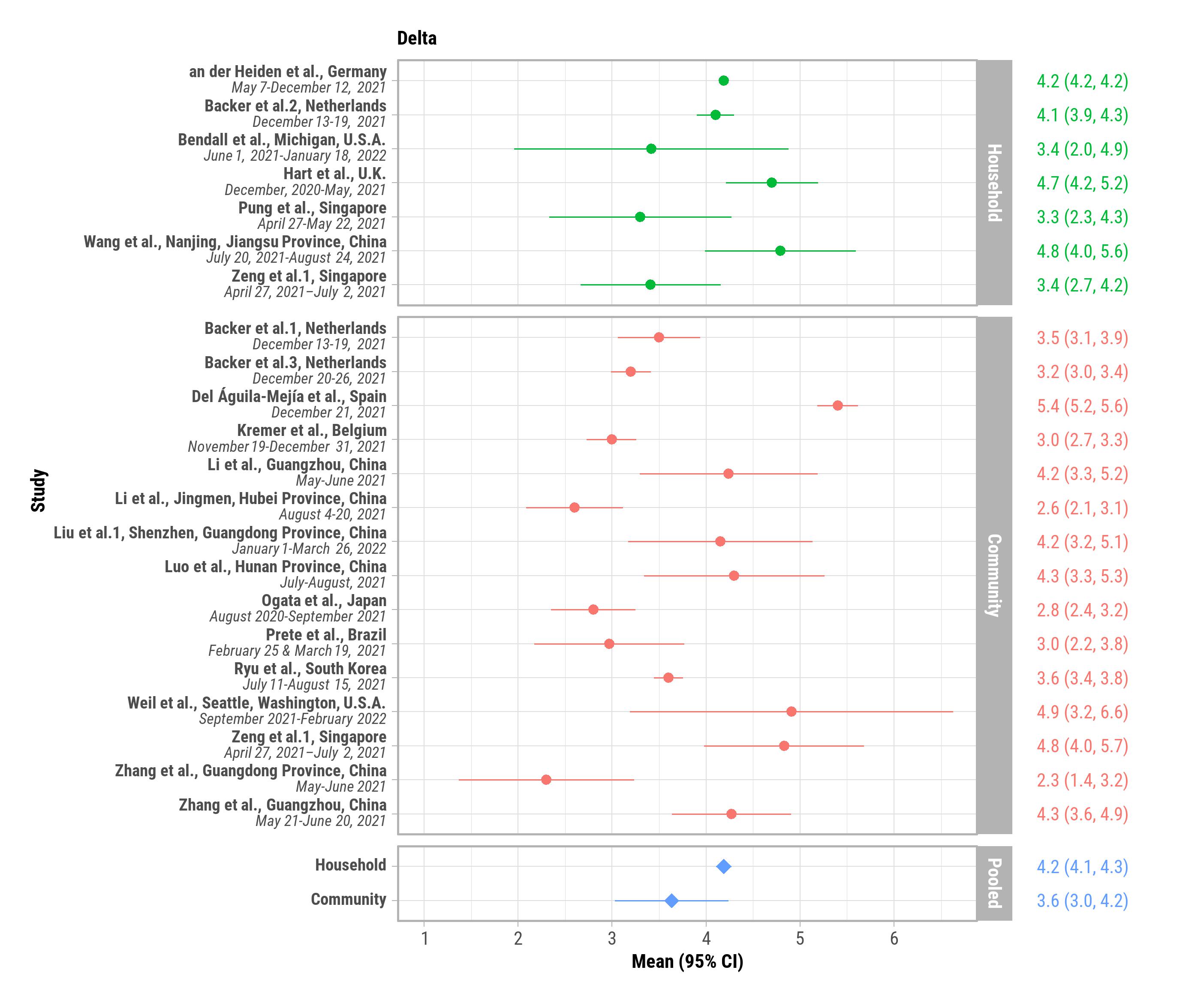


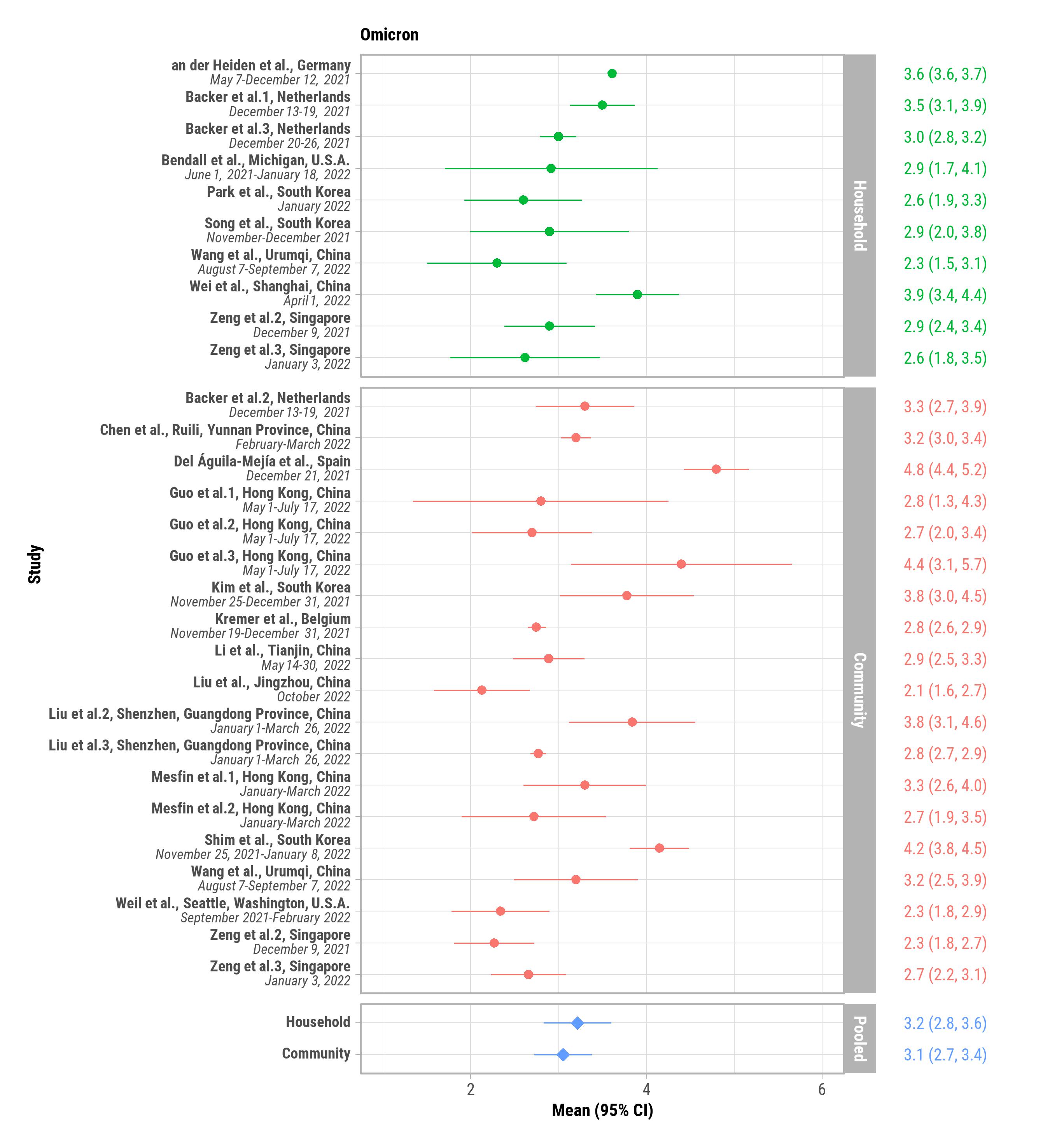


**References**

29. An der Heiden M, Buchholz U. Serial interval in households infected with SARS-CoV-2 variant B. 1.1. 529 (Omicron) is even shorter compared to Delta. Epidemiology & Infection. 2022;150:e165.

30. Backer JA, Eggink D, Andeweg SP, Veldhuijzen IK, van Maarseveen N, Vermaas K, et al. Shorter serial intervals in SARS-CoV-2 cases with Omicron BA. 1 variant compared with Delta variant, the Netherlands, 13 to 26 December 2021. Eurosurveillance. 2022;27(6):2200042.

31. Del Águila-Mejía J, Wallmann R, Calvo-Montes J, Rodríguez-Lozano J, Valle-Madrazo T, Aginagalde-Llorente A. Secondary Attack Rate, Transmission and Incubation Periods, and Serial Interval of SARS-CoV-2 Omicron Variant, Spain. Emerg Infect Dis. 2022 Jun;28(6):1224-8.

32. Kim D, Ali ST, Kim S, Jo J, Lim J-S, Lee S, et al. Estimation of Serial Interval and Reproduction Number to Quantify the Transmissibility of SARS-CoV-2 Omicron Variant in South Korea. Viruses. 2022;14(3):533.

33. Kremer C, Braeye T, Proesmans K, André E, Torneri A, Hens N. Serial Intervals for SARS-CoV-2 Omicron and Delta Variants, Belgium, November 19–December 31, 2021. Emerging Infectious Diseases. 2022;28(8):1699.

34. Li L, Han Z-G, Qin P-Z, Liu W-H, Yang Z, Chen Z-Q, et al. Transmission and containment of the SARS-CoV-2 Delta variant of concern in Guangzhou, China: A population-based study. PLOS Neglected Tropical Diseases. 2022;16(1):e0010048.

35. Li D, Li AE, Li ZQ, Bao Y, Liu T, Qin XR, et al. SARS-CoV-2 Delta Variant in Jingmen City, Hubei Province, China, 2021: Children Susceptible and Vaccination Breakthrough Infection. Front Microbiol. 2022;13:856757.

36. Mefsin YM, Chen D, Bond HS, Lin Y, Cheung JK, Wong JY, et al. Epidemiology of Infections with SARS-CoV-2 Omicron BA.2 Variant, Hong Kong, January-March 2022. Emerg Infect Dis. 2022 Sep;28(9):1856-8.

37. Prete CA, Jr, Buss L, Dighe A, Porto VB, da Silva Candido D, Ghilardi F, et al. Serial interval distribution of SARS-CoV-2 infection in Brazil. Journal of Travel Medicine. 2020;28(2).

38. Pung R, Mak TM, Kucharski AJ, Lee VJ. Serial intervals in SARS-CoV-2 B.1.617.2 variant cases. Lancet. 2021 Sep 4;398(10303):837-8.

39. Ryu S, Kim D, Lim JS, Ali ST, Cowling BJ. Serial Interval and Transmission Dynamics during SARS-CoV-2 Delta Variant Predominance, South Korea. Emerg Infect Dis. 2022 Feb;28(2):407-10.

40. Shim E, Choi W, Kwon D, Kim T, Song Y. Transmission Potential of the Omicron Variant of Severe Acute Respiratory Syndrome Coronavirus 2 in South Korea, 25 November 2021–8 January 2022. Open Forum Infectious Diseases. 2022;9(7).

41. Song JS, Lee J, Kim M, Jeong HS, Kim MS, Kim SG, et al. Serial Intervals and Household Transmission of SARS-CoV-2 Omicron Variant, South Korea, 2021. Emerg Infect Dis. 2022 Mar;28(3):756-9.

42. Wang J, Ma T, Ding S, Xu K, Zhang M, Zhang Z, et al. Dynamic characteristics of a COVID-19 outbreak in Nanjing, Jiangsu province, China. Frontiers in Public Health. 2022;10.

43. Weil AA, Luiten KG, Casto AM, Bennett JC, O’Hanlon J, Han PD, et al. Genomic surveillance of SARS-CoV-2 Omicron variants on a university campus. Nature Communications. 2022;13(1):1-12.

44. Zhang M, Xiao J, Deng A, Zhang Y, Zhuang Y, Hu T, et al. Transmission Dynamics of an Outbreak of the COVID-19 Delta Variant B.1.617.2 - Guangdong Province, China, May-June 2021. China CDC Wkly. 2021 Jul 2;3(27):584-6.

45. Zhang K-x, Hu K, Zou P-a, Luo F, Luo D, Chen Y, et al. Estimation of epidemiological parameters of COVID-19 epidemic caused by Delta variant strain in Guangzhou. Chinese Journal of Disease Control & Prevention. 2022 2022-01-10;26(1):112.

46. Ogata T, Tanaka H, Irie F, Hirayama A, Takahashi Y. Shorter Incubation Period among Unvaccinated Delta Variant Coronavirus Disease 2019 Patients in Japan. International Journal of Environmental Research and Public Health. 2022;19(3):1127.

47. Hart WS, Miller E, Andrews NJ, Waight P, Maini PK, Funk S, et al. Generation time of the alpha and delta SARS-CoV-2 variants: an epidemiological analysis. Lancet Infect Dis. 2022 May;22(5):603-10.

48. Bendall EE, Callear AP, Getz A, Goforth K, Edwards D, Monto AS, et al. Rapid transmission and tight bottlenecks constrain the evolution of highly transmissible SARS-CoV-2 variants. Nature Communications. 2023 2023/01/17;14(1):272.

49. Luo K, Wu Y, Wang Y, Liu Z, Yi L, Zhao S, et al. Transmission Dynamics and Epidemiological Characteristics of the SARS-CoV-2 Delta Variant ― Hunan Province, China, 2021. China CDC Weekly. 2023;5:56.

50. Wei Z, Ma W, Wang Z, Li J, Fu X, Chang H, et al. Household transmission of SARS-CoV-2 during the Omicron wave in Shanghai, China: A case-ascertained study. Influenza and Other Respiratory Viruses. 2023;17(2):e13097.

51. Guo Z, Zhao S, Yam CHK, Li C, Jiang X, Chow TY, et al. Estimating the serial intervals of SARS-CoV-2 Omicron BA.4, BA.5, and BA.2.12.1 variants in Hong Kong. Influenza and Other Respiratory Viruses. 2023;17(2):e13105.

52. Zeng K, Santhya, Soong A, Malhotra N, Pushparajah D, Thoon KC, et al. Serial intervals and incubation periods of SARS-CoV-2 Omicron and Delta variants, Singapore. Emerging Infectious Diseases. 2023.

53. Liu C, Lu J, Li P, Feng S, Guo Y, Li K, et al. A Comparative study on epidemiological characteristics, transmissibility, and pathogenicity of three COVID-19 outbreaks caused by different variants. International Journal of Infectious Diseases.

54. Guo Z, Zhao S, Mok CKP, So RTY, Yam CHK, Chow TY, et al. Comparing the incubation period, serial interval, and infectiousness profile between SARS-CoV-2 Omicron and Delta variants. Journal of Medical Virology.n/a(n/a).

55. Li X, Li R, Lian Q, Wang Y, Gu W, Meng Q. A Small Surge in Incidence of SARS-CoV-2 Omicron Variant in the “Dynamic Zero” Period. Canadian Journal of Infectious Diseases and Medical Microbiology. 2023 2023/03/13;2023:5262117.

56. Chen J, Qiu Y, Shi Y, Wu W, Zheng E, Xu L, et al. Uncovering the Impact of Control Strategies on the Transmission Pattern of SARS-CoV-2—Ruili City, Yunnan Province, China, February–March 2022. China CDC Weekly. 2022;4(46):1032-8.

57. Wang K, Guo Z, Zeng T, Sun S, Lu Y, Wang J, et al. Transmission Characteristics and Inactivated Vaccine Effectiveness Against Transmission of SARS-CoV-2 Omicron BA.5 Variants in Urumqi, China. JAMA Network Open. 2023;6(3):e235755-e.

58. Park E, Choi SY, Lee S, Kim M, Lee K, Lee S, et al. Widespread Household Transmission of SARS-CoV-2 B. 1.1. 529 (Omicron) Variant from Children, South Korea, 2022. Yonsei Medical Journal. 2023;64(5):344-8.

59. Liu T, Xu Q, He S, Ruan D, Huang J, Mao A. Estimation of transmission dynamics parameters for Omicron BA.5.2 variant—based on real-world data. Disease Surveillance. 2023.

60. Weil AA, Luiten KG, Casto AM, Bennett JC, O’Hanlon J, Han PD, et al. Mapping the emergence of SARS-CoV-2 Omicron variants on a university campus. medRxiv. 2022:2022.04.27.22274375.
